# Antagonizing somatostatin receptor subtype 2 and 5 reduces blood glucose in a gut- and GLP-1R–dependent manner

**DOI:** 10.1172/jci.insight.143228

**Published:** 2021-02-22

**Authors:** Sara L. Jepsen, Nicolai J. Wewer Albrechtsen, Johanne A. Windeløv, Katrine D. Galsgaard, Jenna E. Hunt, Thomas B. Farb, Hannelouise Kissow, Jens Pedersen, Carolyn F. Deacon, Rainer E. Martin, Jens J. Holst

**Affiliations:** 1 Department of Biomedical Sciences, Faculty of Health and Medical Sciences, University of Copenhagen, Copenhagen, Denmark.; 2 Novo Nordisk Foundation Center for Basic Metabolic Research, Faculty of Health and Medical Sciences, University of Copenhagen, Copenhagen, Denmark.; 3 Novo Nordisk Foundation Center for Protein Research, Faculty of Health and Medical Sciences, University of Copenhagen, Copenhagen, Denmark.; 4 Department of Clinical Biochemistry, Rigshospitalet, University of Copenhagen, Copenhagen, Denmark.; 5 Lilly Research Laboratories, Lilly, Indianapolis, Indiana, USA.; 6 Department of Endocrinology and Nephrology, Hillerød University Hospital, Hillerød, Denmark.; 7 Medicinal Chemistry, Roche Pharma Research and Early Development, Roche Innovation Center Basel, F. Hoffmann-La Roche Ltd., Basel, Switzerland.

**Keywords:** Endocrinology, Metabolism, Diabetes, G protein&ndash;coupled receptors, Glucose metabolism

## Abstract

Somatostatin (SS) inhibits glucagon-like peptide-1 (GLP-1) secretion in a paracrine manner. We hypothesized that blocking somatostatin subtype receptor 2 (SSTR2) and 5 (SSTR5) would improve glycemia by enhancing GLP-1 secretion. In the perfused mouse small intestine, the selective SSTR5 antagonist (SSTR5a) stimulated glucose-induced GLP-1 secretion to a larger degree than the SSTR2 antagonist (SSTR2a). In parallel, mice lacking the SSTR5R showed increased glucose-induced GLP-1 secretion. Both antagonists improved glycemia in vivo in a GLP-1 receptor–dependent (GLP-1R–dependent) manner, as the glycemic improvements were absent in mice with impaired GLP-1R signaling and in mice treated with a GLP-1R–specific antagonist. SSTR5a had no direct effect on insulin secretion in the perfused pancreas, whereas SSTR2a increased insulin secretion in a GLP-1R–independent manner. Adding a dipeptidyl peptidase 4 inhibitor (DPP-4i) in vivo resulted in additive effects on glycemia. However, when glucose was administered intraperitoneally, the antagonist was incapable of lowering blood glucose. Oral administration of SSTR5a, but not SSTR2a, lowered blood glucose in diet-induced obese mice. In summary, we demonstrate that selective SSTR antagonists can improve glucose control primarily through the intestinal GLP-1 system in mice.

## Graphical abstract

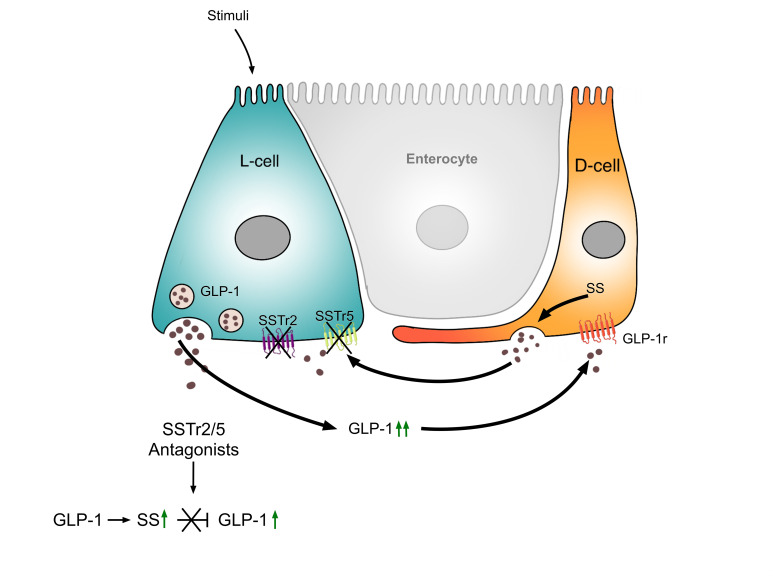

## Introduction

Hormones secreted from the gut are known to be of importance for the regulation of glucose metabolism (1–3), with the incretin hormone glucagon-like peptide-1 (GLP-1) in particular contributing to enhanced postprandial insulin secretion (4–8). Patients with type 2 diabetes have significantly impaired incretin function (9–12), and treatment with stable GLP-1 receptor agonists (GLP-1RAs) or enhancing endogenous levels of active GLP-1 using dipeptidyl peptidase 4 inhibitors (DPP-4is) effectively lower blood glucose levels (13, 14). GLP-1 secretion is regulated by complex mechanism(s). We, and others, have shown that GLP-1 secretion is under paracrine influence from somatostatin (SS) receptor signaling (15–18). Five somatostatin receptor subtypes (SSTR1–SSTR5) exist and prior studies, including our own, have found that SSTR5 in particular is enriched with GLP-1 producing cells (17, 19–21). Furthermore, we found that SSTR5 is a powerful tonic inhibitor of GLP-1 secretion (17). SSTR antagonism may therefore improve glycemic control, and targeting the SSTR5 in vivo as a means to improve glucose tolerance has been suggested (22–25). It is still debated whether the effect of SSTR antagonism on glucose tolerance is mediated by the gut or if there is a direct effect on the endocrine pancreas in which SSTRs are also expressed (22–24, 26–28).

We hypothesize that SSTR5 in particular, and to some degree SSTR2, maintains glucose homeostasis through paracrine regulation of intestinal GLP-1. To address this, we first studied the secretion of glucose-induced GLP-1 secretion in response to blocking either SSTR5 or the less expressed SSTR2 (17, 29) in the isolated perfused mouse intestine. Using different mouse models, we assessed whether SSTR2 and SSTR5 antagonists (SSTR2a and SSTR5a, respectively) improve whole-body glucose metabolism, and we investigated the underlying mechanism(s), including GLP-1 receptor (GLP-1R) signaling. We also combined the SSTR antagonists with a DPP-4i to elucidate whether a further improvement in glycemic control could be obtained if the secreted GLP-1 were protected from degradation. To show that the antagonists acted through gut-derived mechanisms, i.e., through the SS-GLP-1 axis leading to increased endogenous GLP-1 secretion, and not by direct stimulation of the pancreas, we used the isolated perfused mouse pancreas and performed intraperitoneal glucose tolerance tests (IPGTT) in vivo.

## Results

### SSTR5 and SSTR2 antagonism increases glucose-induced GLP-1 secretion with different magnitudes in the isolated perfused WT mouse small intestine.

**Figure 1:**
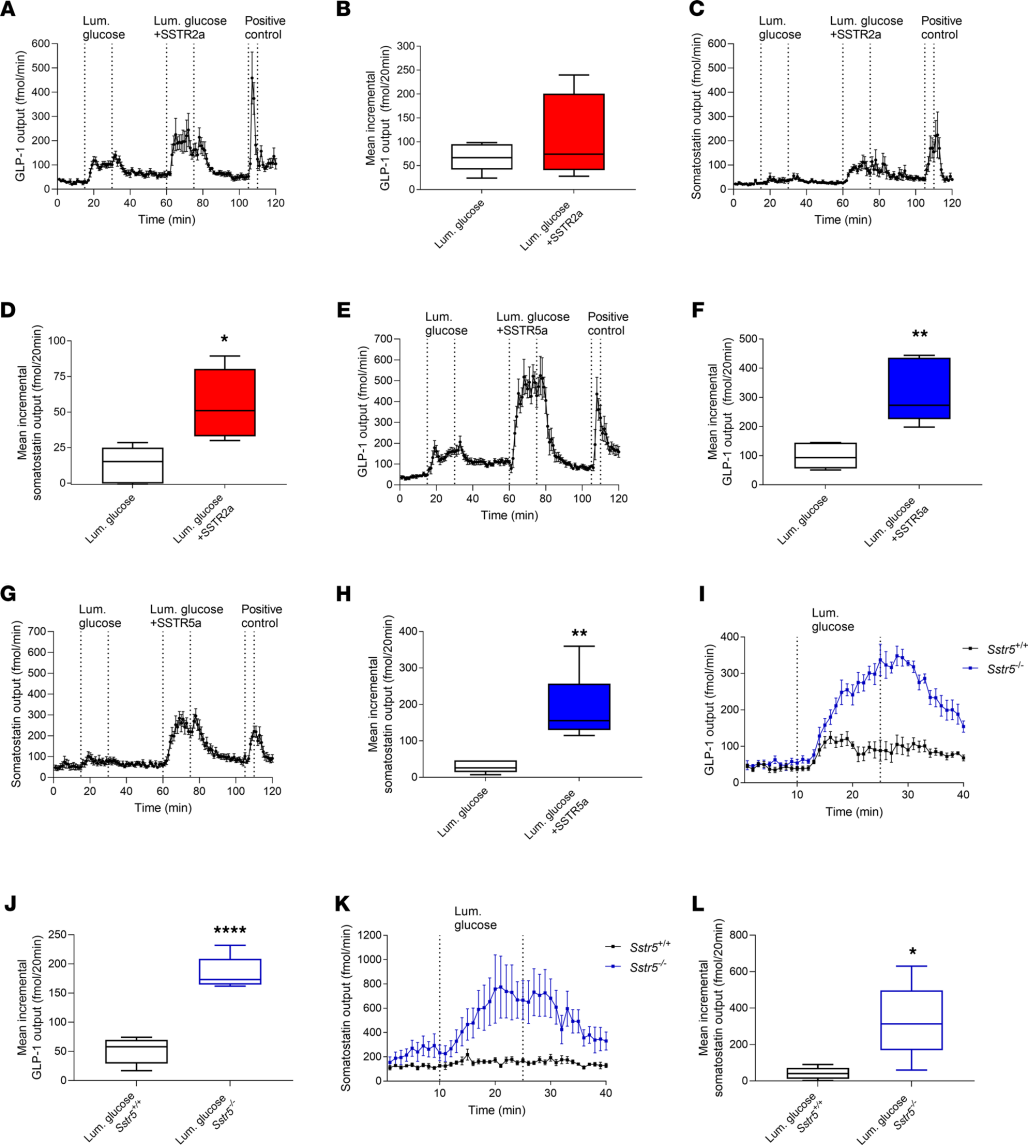
SSTR5a enhances glucose-induced GLP-1 secretion more than SSTR2a in the perfused mouse proximal small intestine. GLP-1 and SS levels in the effluent from the perfused proximal small intestine of nonfasted C57BL/6JRj (**A**–**H**) or S*str5^–/–^* and *Sstr5^+/+^* male mice (**I**–**L**). The intestine preparations were stimulated with luminal glucose (20 % w/v) alone or in combination with a simultaneous intra-arterial infusion of either 1 μM SSTR2a or SSTR5a, where after GLP-1 and SS were measured. (**A**–**D**) GLP-1 and SS output (fmol/min) or mean incremental output (fmol/20 min) in response to glucose and glucose + SSTR2a in C57BL/6JRj mice (GLP-1: *n* = 8, SS: *n* = 5). (**E**–**H**) GLP-1 and SS output (fmol/min) or mean incremental output (fmol/20 min) in response to glucose and glucose + SSTR5a in C57BL/6JRj mice (GLP-1 and SS: *n* = 6). (**I**–**L**) GLP-1 or SS output (fmol/min) or mean incremental output (fmol/20 min) after luminal infusion of glucose in male *Sstr5^–/–^* (blue) or *Sstr5^+/+^* mice (black) (*n* = 5). Bombesin was used as the positive control. Data are presented as the mean ± SEM. Statistical significance was tested by paired *t* test (**B**, **D**, **F**, and **H**) or unpaired *t* test (**J** and **L**). *P < 0.05, **P < 0.01, ***P < 0.001, and ****P < 0001. The box plots show the median and 25th and 75th percentiles and the whiskers represent the smallest and highest value.

The luminal glucose stimulation with vascular SSTR2a tended to increase GLP-1 output in venous effluents compared with glucose stimulation alone (luminal glucose, 67 ± 10 fmol/20 min vs. luminal glucose + SSTR2a, 111 ± 30 fmol/20 min, *P* = 0.15) (Figure 1, A and B). SS secretion followed the same pattern as GLP-1 during SSTR2a infusion, and we found a correlation between SS and GLP-1 concentrations of R^2^ = 0.67 based on average output each minute from 1 minute to 100 minutes (Supplemental Figure 1A; supplemental material available online with this article; https://doi.org/10.1172/jci.insight.143228DS1). In the case of SS secretion, when SSTR2a was combined with luminal glucose the observed increase was significantly higher compared with glucose administration alone (luminal glucose, 13 ± 5 fmol/min vs. luminal glucose + SSTR2a, 55 ± 11 fmol/min *P* = 0.02) (Figure 1, C and D). Intra-arterial infusions of the SSTR5a increased the GLP-1 response to luminal glucose approximately 3-fold (luminal glucose, 98 ± 17 fmol/20 min vs. luminal glucose + SSTR5a, 310 ± 43 fmol/20 min, *P* = 0.0052) (Figure 1, E and F). Based on the fold changes, calculated for the baseline-subtracted glucose-induced mean GLP-1 output versus the mean GLP-1 output after glucose plus SSTR2a or SSTR5a stimulation, we found that SSTR5a increased glucose-induced GLP-1 release more than SSTR2a (fold change for SSTR2a + glucose vs. glucose, 1.75 ± 0.5; fold change for SSTR5a + glucose vs. glucose 3.6 ± 0.6, *P* = 0.03). SS again followed the same pattern as GLP-1, where we found a correlation between SS and GLP-1 secretion, R^2^ = 0.89 (Supplemental Figure 1B), and the increase in SS output was significantly higher when SSTR5a was combined with luminal glucose compared with glucose administration alone (luminal glucose, 28 ± 6 fmol/min vs. luminal glucose + SSTR5a, 190 ± 37 fmol/min *P* = 0.0045) (Figure 1, G and H).

We then investigated whether glucose-induced GLP-1 secretion would be increased in a mouse model lacking SSTR5 (*Sstr5^–/–^* mice). Glucose-induced GLP-1 output in *Sstr5^–/–^* mice increased more than 3-fold compared with that of WT littermates (*Sstr5^+/+^* mice, 51 ± 10 fmol/min vs. *Sstr5^–/–^* mice, 184 ± 13 fmol/min, *P* < 0.0001) (Figure 1, I and J). SS outputs were likewise significantly higher upon glucose stimulation in *Sstr5^–/–^* mice compared with *Sstr5^+/+^* mice (*Sstr5^+/+^*, 41.3 ± 15 fmol/20 min, *Sstr5^–/–^* mice, 329.2 ± 91 fmol/20 min, *P* = 0.0017) (Figure 1, K and L) and correlated with the GLP-1 response (*Sstr5^+/+^* R^2^ = 0.51, *Sstr5^–/–^* R^2^ = 0.83) (Supplemental Figure 1, C and D).

### SSTR5 and SSTR2 antagonism lower blood glucose during an OGTT via a gut-dependent mechanism.

**Figure 2:**
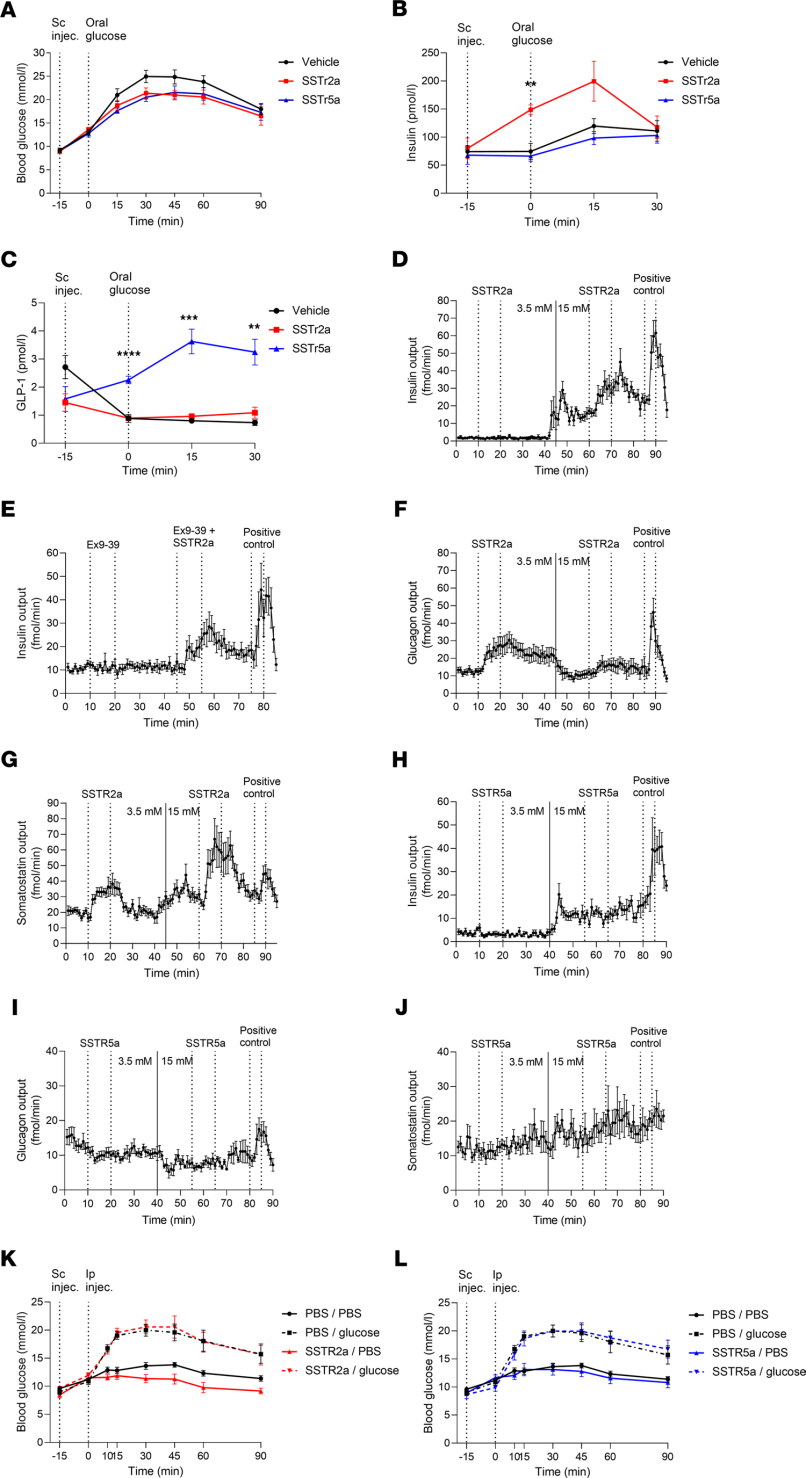
SSTR2a and SSTR5a lower blood glucose in vivo. **During hyperglycemia, in the perfused pancreas, SSTR2a increases insulin whereas SSTR5a does not**. (**A**–**C**) Plasma blood glucose (mmol/L), insulin levels (pmol/L), and GLP-1 levels (pmol/L) after male C57BL/6JRj mice received vehicle (black), 4 mg/kg SSTR2a (red), or SSTR5a (blue) by s.c. injection 15 minutes before an oral glucose load (*n* = 8). (**D**–**J**) Insulin, glucagon, and insulin levels after the pancreas was perfused with a perfusion buffer at low-glucose concentration (3.5 mM) from 0 to 40 minutes, after which the buffer was exchanged to a high-glucose-containing buffer (15 mM) for the rest of the experiment (**D** and **F**–**J**) or at a constant concentration of 15 mM (**E**). 1 μM SSTR2a, SSTR5a, or Ex9–39 was added to the arterial perfusate via a side-arm. 10 mM arginine was used as positive control at the end of each perfusion experiment (*n* = 6). (**K** and **L**) Male mice received s.c. injections of vehicle (PBS), 4 mg/kg SSTR2a, or SSTR5a at time –15 minutes, and at time 0 minutes they received the i.p. injection of glucose or PBS. (**K**) Blood glucose (mmol/L) levels after the following injections: PBS s.c. at –15 minutes and i.p. PBS at 0 minutes (black line), s.c. PBS at time –15 minutes and i.p. glucose at 0 minutes (black dashed line), s.c. SSTR2a at –15 minutes and i.p. PBS at 0 minutes (red line), or s.c. SSTR2a at –15 minutes and i.p. glucose at 0 minutes (red dashed line) (*n* = 8). (**L**) The same as in **K**, but with SSTR5a, represented in blue. SSTR5a s.c. at –15 minutes and i.p. PBS (blue line), i.p. glucose at 0 minutes and s.c. SSTR5a at –15 minutes (blue dashed line) (*n* = 8). Data are presented as the mean ± SEM. Statistical significance at specific time points was assessed by 2-way ANOVA followed by Tukey post hoc analysis to correct for multiple testing in vivo and by paired *t* test in the perfusion experiments. **P < 0.01, ***P < 0.001, ****P < 0.0001.

To investigate whether the increase in glucose-induced GLP-1 secretion, caused by SSTR2a and SSTR5a in the perfused mouse intestine, would affect glucose tolerance during an oral glucose tolerance test (OGTT) in vivo, the antagonists were administered s.c. 15 minutes before an oral glucose load in male mice. Similar experiments were carried out in female mice, revealing no sex-specific variations (Supplemental Figure 2, A–C). Both SSTR2a and SSTR5a significantly lowered glucose concentrations compared with vehicle (iAUC_0–90 min_ vehicle, 794 ± 55 mmol/L × min vs. SSTR2a 508 ± 69 mmol/L × min, *P* = 0.01 and vehicle vs. SSTR5a, 579 ± 73 mmol/L × min, *P* = 0.03) (Figure 2A). When comparing the iAUC_0–30_ there was no overall effect on insulin levels of either SSTR2a or SSTR5a compared with vehicle (vehicle vs. SSTR2a, *P* = 0.6 and vehicle vs. SSTR5a, *P* = 0.9), although SSTR2a did increase insulin levels significantly after 15 minutes of administration (at time 0 min) compared with vehicle (*P* = 0.002) (Figure 2B). Total GLP-1 levels were significantly higher in SSTR5a-treated mice compared with those treated with vehicle at time 0 minutes (*P* < 0.0001), 15 minutes (*P* < 0.0008), and 30 minutes (*P* < 0.004) (Figure 2C), whereas no significant effect was observed in SSTR2a-treated mice (*P* = 0.2).

We then investigated whether an additional improvement in glucose tolerance could be obtained by combining SSTR2a and SSTR5a during an OGTT. When combining the 2 antagonists, an additive improvement of glucose tolerance compared with vehicle was observed, and the combination significantly improved both blood glucose levels and insulin secretion compared with vehicle (Supplemental Figure 2, D and E).

To investigate the direct pancreatic effects of SSTR5a and SSTR2a, we used the isolated perfused pancreas model in male mice and infused the antagonists by intra-arterial infusion at low (3.5 mM) and high (15 mM) glucose.

SSTR2a did not affect the mean insulin output compared with the mean preceding baseline at low glucose (*P* = 0.5), whereas an increase from the preceding baseline period was observed at high glucose (*P* = 0.005) (Figure 2D). This increase in insulin was unchanged when the SSTR2a was combined with the GLP-1R antagonist exendin9–39 (Ex9–39), where no difference between the 2 peaks was found based on incremental area under the curve (iAUC) (*P* = 0.16) (Figure 2E). SSTR2a significantly increased mean glucagon output at low glucose (*P* = 0.005), and a significant, albeit minor, increase was observed at high glucose (*P* = 0.04) (Figure 2F). SSTR2a significantly increased the mean SS output both at low and high glucose levels (*P* = 0.0007 and *P* = 0.02, respectively) (Figure 2G).

SSTR5a did not affect insulin secretion at low or high glucose (*P* = 0.4 and *P* = 0.9, respectively) (Figure 2H). A small significant decrease in glucagon secretion was observed at low glucose (*P* = 0.02), although the drop was not meaningful, decreasing from 12 ± 1 fmol/min to 10 ± 1 fmol/min, while no effect was observed on glucagon secretion at high glucose (*P* = 0.5) (Figure 2I). Compared with the preceding baseline period, there was no effect of the antagonist on SS levels at either low or high glucose (*P* = 0.6 and *P* = 0.5, respectively) (Figure 2J).

To corroborate our hypothesis, that SSTR5 antagonism in particular lowers blood glucose through gut-derived mechanisms and not through direct stimulation of pancreatic hormones, we administered SSTR2a and SSTR5a by s.c. injections 15 minutes before the i.p. injection of glucose or PBS, thereby avoiding stimulating intestinally derived GLP-1. When glucose was administered i.p. instead of orally, neither antagonist had any effect on blood glucose levels compared with the control group (iAUC_0–90 min_ s.c. PBS/i.p. glucose, 627 ± 83 mmol/L × min. vs. s.c. SSTR2a/i.p. glucose, 570 ± 103 mmol/L × min, *P* = 0.5, and s.c. PBS/i.p. glucose vs. s.c. SSTR5a/i.p. glucose, 735 ± 67 mmol/L × min, *P* = 0.4) (Figure 2, K and L).

### SSTR5a and SSTR2a lower blood glucose in a GLP-1R–dependent manner and when combined with a DPP-4i further improve glucose tolerance.

**Figure 3:**
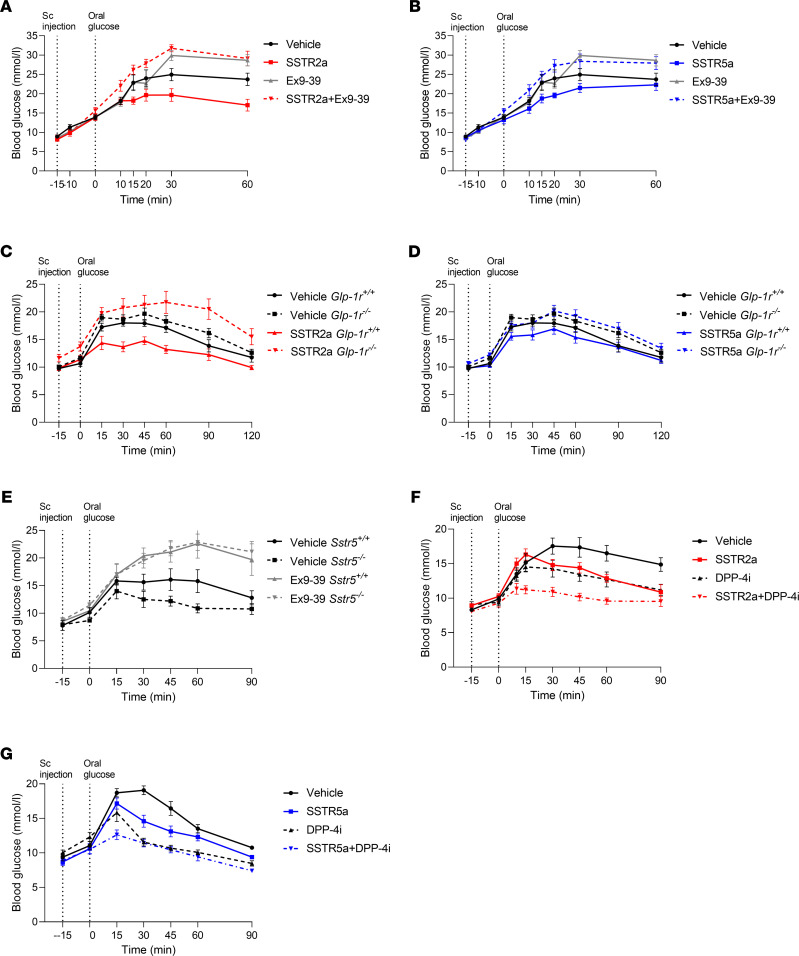
The glucose-lowering effect of SSTR2a and SSTR5a is GLP-1R dependent. (**A**) Blood glucose levels (mmol/L) in C57BL/6JRj mice receiving vehicle (black line), 4 mg/kg SSTR2a (red line), 4 mg/kg Ex9–39 (gray line), or SSTR2a + Ex9–39 (dashed red line) s.c. 15 minutes before an oral glucose load (*n* = 5–8). (**B**) The same as **A** but for 4 mg/kg SSTR5a (blue), 4 mg/kg SSTR5a + 4 mg/kg Ex9–39 (dashed blue line), *n* = 5–8. (**C**) Blood glucose levels (mmol/L) in *Glp-1r^–/–^* or *Glp-1r^+/+^* mice after vehicle or 4 mg/kg SSTR2a. *Glp-1r^+/+^* receiving vehicle (black line), *Glp-1r^–/–^* receiving vehicle (black dashed line), *Glp-1r^+/+^* receiving 4 mg/kg SSTR2a (red line), *Glp-1r^–/–^* receiving 4 mg/kg SSTR2a (dashed red line), *n* = 8–13. (**D**) The same as in **C** but for 4 mg/kg SSTR5a indicated with blue. *Glp-1r^+/+^* receiving 4 mg/kg SSTR5a (blue line), *Glp-1r^–/–^* receiving 4 mg/kg SSTR5a (dashed blue line), *n* = 10–13. (**E**) Blood glucose levels (mmol/L) in *Sstr5^–/–^* or *Sstr5^+/+^* mice receiving vehicle or 4 mg/kg Ex9–39. *Sstr5^+/+^* receiving vehicle (black line), *Sstr5^–/–^* receiving vehicle (black dashed line), *Sstr5^+/+^* receiving 4 mg/kg Ex9–39 (gray line), *Sstr5^–/–^* receiving 4 mg/kg Ex9–39 (gray dashed line) (*n* = 4–5). (**F**) Blood glucose levels (mmol/L) after administration of vehicle (black line), 4 mg/kg SSTR2a (red line), 120 mg/kg DPP-4 (dashed black line), and a combination of SSTR2a and DPP-4i (red dashed line) (*n* = 8). (**G**) The same as in **F** but for 8 mg/kg SSTR5a (blue line) and SSTR5 + DPP-4i (blue dashed line) (*n* = 6–8). Data are shown as the mean ± SEM, and significance was evaluated based on iAUC by 1-way ANOVA followed by the Holm-Sidak post hoc analysis to correct for multiple testing.

To evaluate whether the improved glucose tolerance observed when SSTR2 and SSTR5 are antagonized is GLP-1R dependent, we combined them with Ex9–39, a well-characterized GLP-1R antagonist. The presence of Ex9–39 abolished the glucose-lowering effect of both SSTR2a and SSTR5a during an OGTT (iAUC_0–60 min_ of SSTR2a, 295 ± 177 mmol/L × min vs. SSTR2a + Ex9–39, 717 ± 80 mmol/L × min *P* < 0.0001; SSTR5a, 361 ± 82 mmol/L × min vs. SSTR5a + Ex9–39, 624 ± 69 mmol/L × min *P* = 0.013) (Figure 3, A and B).

In line with the effect of GLP-1R antagonism, similar findings were observed in *Glp-1r ^–/–^* mice in which the lowering of blood glucose by SSTR2a and SSTR5a was significantly diminished compared with that observed in *Glp-1r^+/+^* animals (iAUC_0-120 min_ SSTR2 in *Glp-1r^+/+^*, 177.8 ± 28 mmol/L × min vs. SSTR2 in *Glp-1r ^–/–^*, 421 ± 78 mmol/L × min, *P* = 0.01; SSTR5 in *Glp-1r^+/+^*, 265 ± 29 mmol/L × min vs. SSTR5 in *Glp-1r^–/–^*, 435 ± 63 mmol/L × min, *P* = 0.04) (Figure 3, C and D). In this subset of experiments, no significant difference between the SSTR5a and vehicle group in *Glp-1r^+/+^* was observed (*P* = 0.4). This could be due to the rather low dose of the antagonist that was used (4 mg/kg), resulting in variability of the antagonistic effect. In follow-up studies, we observed that increasing the dose as well as changing the route of administration to oral gavage markedly increased the effect of the SSTR5a on blood glucose (Supplemental Figure 3).

As *Sstr5^–/–^* mice had increased glucose-induced GLP-1 release compared with WT littermates when their intestine was perfused (Figure 1I), we investigated whether they might have improved glucose control in vivo and whether this could be abolished by adding Ex9–39 during an OGTT. No significant difference in blood glucose levels was observed when comparing *Sstr5^–/–^* with *Sstr5^+/+^* (iAUC_0–90 min_ vehicle *Sstr5^+/+^*, 424.4 ± 118 mmol/L × min vs. vehicle *Sstr5^–/–^*, 269.6 ± 51 mmol/L × min, *P* = 0.28) (Figure 3E), which otherwise has been observed by Farb et al. (23). That only a numerical difference is observed here is most likely due to the low number of mice available in the present study. When the 2 groups received Ex9–39, thus removing the effect of GLP-1, blood glucose levels were significantly impaired compared with their respective vehicles (iAUC_0–90 min_ *Sstr5^+/+^* vehicle, 424.4 ± 118 mmol/L × min vs. *Sstr5^+/+^* + Ex9–39, 820.1 ± 144 mmol/L × min, *P* = 0.03; *Sstr5^–/–^* vehicle, 269.6 ± 51 mmol/L × min vs. *Sstr5^–/–^* + Ex9–39, 763.3 ± 82.7 mmol/L × min, *P* = 0.006) (Figure 3E).

Having shown that SSTR2a and SSTR5a lower blood glucose by potentiating glucose-induced GLP-1 secretion (Figure 3, A–D), we investigated whether an improved glycemic control could be obtained when GLP-1 degradation is prevented, namely by combining the SSTR antagonists with the DPP-4i. Animals receiving the combination of SSTR2a and DPP-4i showed an additive effect on glucose levels compared with vehicle (iAUC_0–90 min_: vehicle, 606 ± 76 mmol/L × min vs. DPP-4i, 323 ± 58 mmol/L × min, *P* = 0.003; vehicle vs. SSTR2a, 321 ± 38 mmol/L × min, *P* = 0.04; vehicle vs. SSTR2a + DPP-4i, 144 ± 30 mmol/L × min, *P* < 0.0001) (Figure 3F). The combination of SSTR5a and DPP-4i likewise improved blood glucose levels in an additive manner compared with vehicle (iAUC_0–90 min_: vehicle, 380 ± 52 mmol/L × min vs. DPP-4i, 176 ± 36 mmol/L × min, *P* = 0.002; vehicle vs. SSTR5a, 217 ± 28 mmol/L × min, *P* = 0.012; vehicle vs. SSTR5 + DPP-4i, 146.2 ± 26.8 mmol/L × min, *P* = 0.0009) (Figure 3G). However, no difference in iAUC was observed between SSTR5a alone and SSTR5a + DPP-4i (*P* = 0.3), but upon mixed effects analysis, a significant difference between the 2 groups was observed at time 15 minutes (*P* = 0.004).

### SSTR5a is more effective at stimulating glucose-induced GLP-1 secretion in the perfused intestine as well as improving glucose tolerance in vivo than SSTR2a in DIO and control mice.

Using diet-induced obese (DIO) mice, we evaluated whether the effect of antagonizing SSTR5 and SSTR2 also translates in a model of disease. The effect of antagonizing SSTR2a and SSTR5a on glucose-induced GLP-1 and SS secretion was investigated in the proximal perfused mouse intestine and their effects on glucose tolerance were evaluated in vivo.

**Figure 4:**
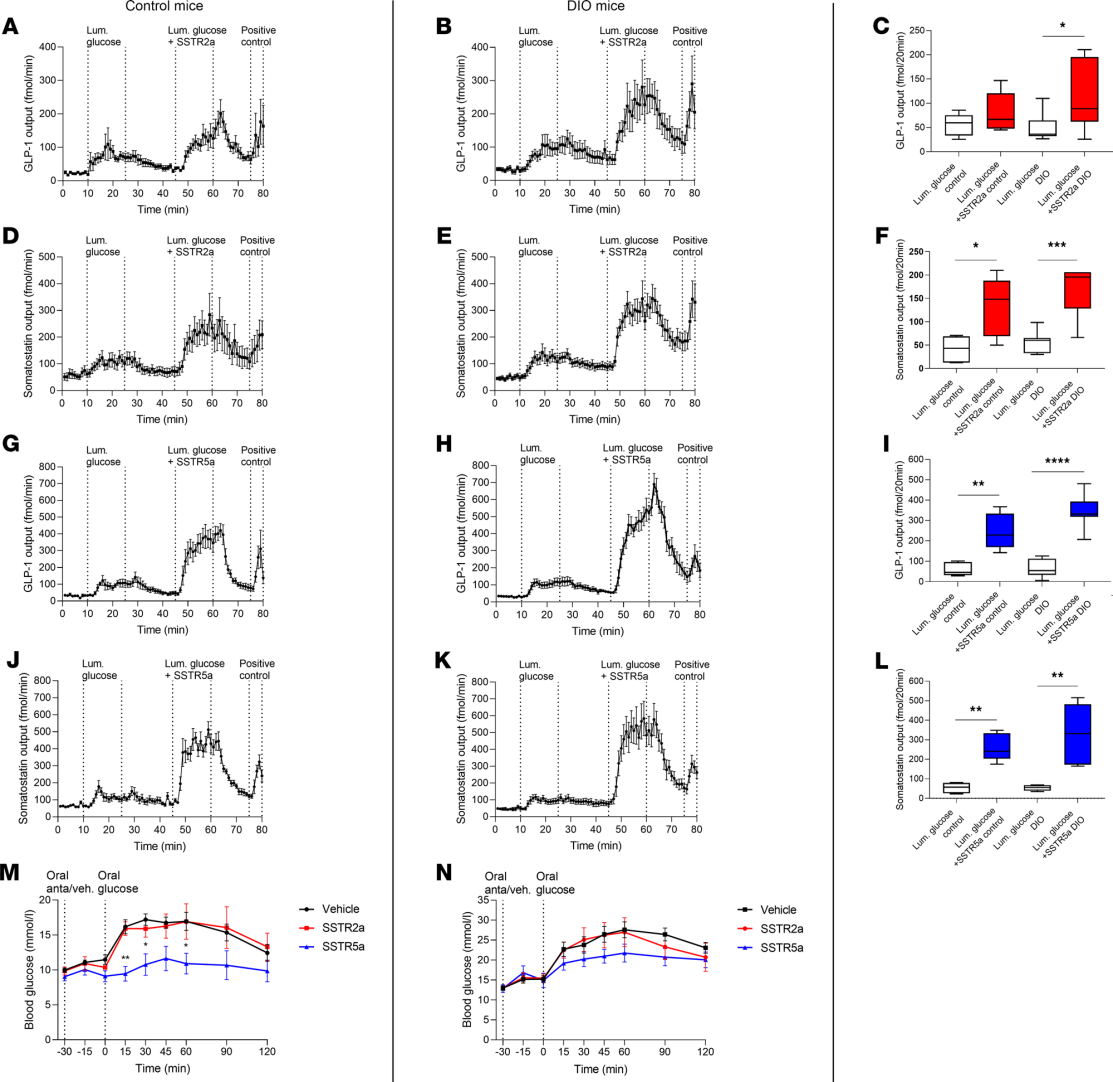
In proximal perfused intestine of DIO and control mice, SSTR5a stimulates glucose-induced GLP-1 secretion more than SSTR2a, and SSTR5a improves glucose tolerance when applied orally in DIO mice. (**A**–**L**) GLP-1 an SS output (fmol/min) or mean incremental output (fmol/20 min) in proximal intestinal perfusions of control and DIO mice. The intestine was stimulated with luminal glucose alone or in combination with simultaneous intra-arterial infusion of 1 μM SSTR2a or SSTR5a. (**A**) *n* = 7, (**B**) *n* = 5, (**D**) *n* = 7, (**E**) *n* = 6, (**G**) *n* = 7, (**H**) *n* = 5, (**J**) *n* = 7, and (**K**) *n* = 4. (**M** and **N**) In vivo studies in control and DIO mice undergoing an OGTT after oral administration of vehicle (black), 50 mg/kg SSTR2a (red), or SSTR5a (blue) 30 minutes before oral glucose. (**M**) Blood glucose levels (mmol/L) (*n* = 5–6, control mice). (**N**) Same as in **M**, but in DIO mice (*n* = 4). Data are presented as the mean ± SEM. Statistical significance at specific time points was assessed by 2-way ANOVA followed by Tukey post hoc analysis to correct for multiple testing (**M**) and by paired *t* test (**C**, **F**, **I**, and **L**), *P < 0.05, **P < 0.01, ***P < 0.001, ****P < 0.0001. The box plots show the median and 25th and 75th percentiles, and the whiskers represent the smallest and highest values.

Intra-arterial infusions of SSTR2a did not significantly increase glucose-induced GLP-1 secretion in control mice (luminal glucose, 55.5 ± 20.3 fmol/20 min vs. luminal glucose + SSTR2a, 80.6 ± 18.7 fmol/20 min, *P* = 0.26). In DIO mice, SSTR2a infusion led to a 2-fold increase in glucose-induced GLP-1 secretion (luminal glucose, 57.3 ± 11 fmol/20 min vs. luminal glucose + SSTR2a, 112 ± 26.6 fmol/20 min, *P* = 0.029) (Figure 4, A–C). SS secretion increased during the intra-arterial infusions of SSTR2a compared with luminal glucose infusion alone (luminal glucose vs. SSTR2a + glucose *P* = 0.01) (Figure 4, D–F). SSTR5a again potentiated glucose-induced GLP-1 secretion the most, with a 4-fold increase in control mice (luminal glucose, 60.9 ± 14.9 fmol/20 min vs. luminal glucose + SSTR5a, 246.5 ± 39.61 fmol/20 min, *P* = 0.0061) and a 5-fold increase in DIO mice (luminal glucose, 65.9 ± 16.7 fmol/20 min vs. luminal glucose + SSTR5a, 344.1 ± 31.54 fmol/20 min, *P* < 0.0001) (Figure 4, G–I). During intra-arterial infusions of SSTR5a, SS secretion increased compared with glucose infusion alone (glucose vs. SSTR5a + glucose *P* = 0.005) (Figure 4, J–L).

The effect of the SSTR2a and SSTR5a on blood glucose during an OGTT in DIO and control mice was evaluated by s.c. injection of the antagonists, 15 minutes before an oral glucose load. However, there was no effect on glucose tolerance after SSTR2a and SSTR5a in DIO mice, whereas an effect was observed in control mice (Supplemental Figure 4). This may be due to the route of administration; therefore, we used oral administration of the antagonists in the subsequent experiments. Surprisingly, the SSTR2a did not affect glucose tolerance compared with vehicle in DIO or control mice (iAUC_0-120 min_ control: vehicle vs. SSTR2a, *P* = 0.9; DIO: vehicle vs. SSTR2, *P* = 0.7) (Figure 4, M and N). In DIO mice, SSTR5a significantly improved glucose tolerance during an OGTT with a trend (*P* = 0.07) being observed in control mice (iAUC_0-120 min_ control: vehicle, 561 ± 113 mmol/L × min, *n* = 8 vs. SSTR5a, 167 ± 32 mmol/L × min, *n* = 5; DIO: vehicle, 1141 ± 106 mmol/L × min, *n* = 9, vs. SSTR5a, 654 ± 106 mmol/L × min, *n* = 9, *P* = 0.02) (Figure 4, M and N).

## Discussion

We and others have documented *Sstr2* and *Sstr5* expression on the GLP-1–secreting L cells, where *Sstr5* expression clearly exceeded the level of *Sstr2* expression (17, 29). Expression of these receptors has also been reported in the islets of the pancreas, both on α and β cells (26, 27, 30, 31), and previous studies have shown that SSTR5 antagonism can improve blood glucose levels (22–24). Whether this is due to a gut-mediated effect, a direct effect on the pancreas, or a combination was unclear. Most studies have focused on the effect of SSTR5, whereas the effect of SSTR2 antagonism on glucose tolerance has barely been touched upon. In this study, we demonstrate that antagonizing SSTR2 and SSTR5 pharmacologically improved glucose tolerance by potentiating glucose-induced GLP-1 from the small intestine.

We used the isolated perfused mouse proximal intestine to investigate the paracrine actions of SSTRs on glucose-induced GLP-1 secretion. The perfused organ models preserve the circulatory system and the paracrine relationships; they minimize degradation of hormones, eliminate interference from other organs and circulating factors, and therefore allow accurate studies of the local secretory patterns of hormone secretion, precisely as they occur in vivo, but where such measurements cannot be performed due to degradation of hormones in the circulation and limited amount of plasma (32).

In the perfused mouse proximal intestine, glucose-induced GLP-1 secretion tended to be increased by SSTR2a, but the SSTR5a was by far the most effective stimulus, resulting in a 3-fold increase in GLP-1 secretion in C57BL/6 mice, and a similar, enhanced secretion of GLP-1 was observed in *Sstr5^–/–^* mice when glucose was infused intraluminally. Common to all perfusion studies, SS secretion followed a similar pattern as GLP-1 secretion, which is in line with our previous finding that SS secretion is dependent on GLP-1 stimulation (17), as adding the GLP-1R antagonist (Ex9–39) with SSTR5a completely abolished the secretion of SS. This, therefore, suggests that the increased SS secretion, when antagonizing the SSTR5, is brought about by an effect of the antagonists on the L cell rather than by the disruption of an autocrine feedback on the D cell (17). We expect that the same applies to the SSTR2a, although this has not yet been tested.

Based on these studies, we expected that the SSTR5a would improve glucose tolerance in a gut-derived, GLP-1–dependent manner during an OGTT in vivo, whereas the SSTR2a would be less effective. Surprisingly, we found that both SSTR5a and SSTR2a enhanced glucose tolerance equally during an OGTT in WT male and female mice.

Measuring total GLP-1 levels in vivo revealed that the effect elicited by the SSTR5a on glucose tolerance most likely is due to increases in GLP-1 secretion, which is in line with the findings by Farb et al. (23). Even before glucose was administered (at time 0 min), GLP-1 levels were elevated, confirming the strong tonic inhibitory effect exerted by the SSTR5 on GLP-1 secretion, even in the basal state (16, 17). No effect on circulating GLP-1 levels was observed for SSTR2a. It should be noted that we were unable to detect increases in GLP-1 levels in the vehicle group after glucose administration in male mice and, surprisingly, we observed a drop from –15 to 0 minutes, whereas an increase was observed in female mice. Additionally, we saw a lower basal secretion of GLP-1 in response to SSTR5a in male mice compared with female mice and there was a difference in the response to glucose. An accurate estimation of low levels of endogenous GLP-1 secretion in mice has proven to be challenging due to the pronounced activity of neutral endopeptidase 24.11 (33, 34), and there is currently no way of providing more accurate measurements of GLP-1 secretion in mice in vivo. With the applied sandwich ELISA (the most sensitive available), it is most likely only a small fraction of the true GLP-1 release that is detected. However, the possibility that there is a genuine sex difference cannot be ruled out, which requires further investigation.

We have previously characterized the antagonistic properties of SSTR2a used for this study, which turned out to be less specific than first anticipated, i.e., antagonizing also SSTR1, SSTR3, and SSTR4 to a variable degree (17). Furthermore, a study by Hocart et al. showed that the SSTR2a additionally can bind to the SSTR5 (35), although we found no inhibition of SSTR2a on SSTR5 in our previous study. The SSTR5a, on the other hand, was found to be selective to its receptor (17). The relative nonselectivity of the applied SSTR2a, possibly targeting multiple glucose regulating pathways in addition to the GLP-1–producing L cells, could explain why SSTR2a decreases blood glucose in vivo (17). Thus, SSTR2a may cause stimulation of pancreatic hormone release in vivo, as expression of *Sstr1*, *Sstr2*, and *Sstr3* has been found in the pancreatic islets (36, 37). Expression of *Sstr5* has likewise been reported in rodent β cells and SSTR5 has been suggested to be the main regulator of insulin secretion in mice (27, 28, 30, 38). However, in our study in the perfused mouse pancreas, there was no effect of SSTR5 antagonism on insulin secretion, either at low or high glucose levels, consistent with a study involving isolated mouse islets (23). In contrast, SSTR2 antagonism increased insulin during hyperglycemia as well as increased glucagon secretion during hypoglycemia. An effect of SSTR2a on glucagon secretion during hypoglycemia has previously been shown in streptozotocin-induced diabetic rats and has been suggested as a target to restore the glucagon response to hypoglycemia in type 1 diabetes (39–42). The effect of SSTR2a on insulin secretion during hyperglycemia has been studied in SSTR2-knockout mice and in isolated cells but revealed only weak effects on insulin secretion (30, 43). In contrast, our results from the perfused pancreas demonstrated that SSTR2a can increase insulin secretion during hyperglycemia.

It has been suggested that secretion of α cell–derived GLP-1 plays a role in glucose homeostasis in mice (44). GLP-1 is a product of posttranslational processing of proglucagon, which is produced in intestinal L cells as well as in pancreatic α cells. Since the SSTR2a elicited a minor increase in glucagon concentrations during hyperglycemia in the perfused pancreas, it could be speculated that α cell–derived GLP-1 would be released in parallel, which could stimulate insulin release. However, the increase in insulin secretion by the SSTR2a was still evident when combined with Ex9–39, suggesting that the effects of SSTR2a on insulin levels do not involve the GLP-1R in the isolated perfused pancreas.

Surprisingly, our in vivo data show that when SSTR2a is given in combination with i.p. glucose (circumventing intestinal hormone release), the effect of SSTR2a on glucose tolerance was completely blunted. The same was observed for the SSTR5a, suggesting that both antagonists improve glucose tolerance in vivo through a gut-derived mechanism and not by directly affecting pancreatic secretion. It should be noted that even though GLP-1 levels were modestly elevated after SSTR5a injection during fasting (time 0 min, Figure 2C), this was not associated with any improvement in glycemic control at time 0 minutes during the IPGTT experiments. A possible explanation could be that the elevation by SSTR5a in the fasting state is too small to significantly influence insulin/glucagon secretion and thereby glucose tolerance during the IPGTT. Furthermore, we also observed increased levels of insulin during an OGTT at fasting when SSTR2a was injected. Again, however, blood glucose levels during the IPGTT at time 0 minutes were not affected. Insulin levels during the IPGTT were measured (data not shown) but did not show any elevations after the SSTR2a injection. The lack of insulin response could be related to a stress reaction to handling, as activation of the sympathetic nerves is known to markedly inhibit glucose-induced insulin release (45, 46).

The GLP-1 dependency of the improvement of glucose tolerance by SSTR2 and SSTR5 antagonism in vivo was also evident when WT mice received the SSTR antagonists together with Ex9–39, which completely abolished their beneficial effects on glucose tolerance. Conversely, blocking GLP-1R with the same antagonist in *Sstr5^–/–^* mice worsened their otherwise improved glucose tolerance, which seemed more pronounced in *Sstr5^–/–^* mice receiving Ex9–39 than in the *Sstr5^+/+^* mice receiving Ex9–39. In *Glp-1r^–/–^* mice, neither SSTR2a nor SSTR5a influenced blood glucose levels.

Consistent with the notion that SSTR2a and SSTR5a act through GLP-1, we combined the SSTR2a and SSTR5a with a DPP-4i and saw an additional improvement of glucose tolerance during an OGTT, in agreement with findings previously reported by Farb et al. regarding SSTR5a (23). It should be noted that when DPP-4 is inhibited, we cannot exclude the contribution of other DPP-4 substrates, such as GIP or peptide YY (14, 47). However, how SSTR2 and SSTR5 antagonism affect these hormones are still unknown.

Having demonstrated that application of SSTR2a and SSTR5a improves glucose tolerance in WT mice, we next investigated their effect in DIO mice. In perfused intestine from these animals (and controls), both SSTR2a and SSTR5a increased glucose-induced GLP-1 secretion. When the antagonists were applied by s.c. injection in vivo, they improved glucose tolerance in WT mice, whereas no effect was seen in DIO mice. We changed the route of administration from s.c. to oral gavage after the SSTR5a significantly improved glucose tolerance in both control and DIO mice, supporting findings from earlier studies (22, 23). However, the SSTR2a was incapable of decreasing blood glucose in control and DIO mice. That the SSTR2a did work in control mice by s.c. injection could suggest that this particular SSTR2a is has poor oral availability; however, this needs further investigation.

Overall, our findings would suggest that clinical development of SSTR5 antagonism, rather than SSTR2 antagonism, may be attractive to achieve improved glycemic control. However, care should be taken when directly comparing 2 antagonists based on a single dose in vivo. Some of our follow-up studies using higher doses of the SSTR5a and changing the route of application to oral instead of s.c. administration showed even clearer effects of the SSTR5a on blood glucose. Thus, a more detailed characterization of the 2 antagonists in vivo, as carried out in the perfusion studies (17), should be performed. However, the wide expression of SSTR2 argues against antagonism for this receptor for future pharmacological development, as this may influence many systems; importantly, the *Sstr2* has been found to be highly expressed in certain types of cancers (48–50). SSTR5, on the other hand, is much more discretely expressed with particularly high expression levels in the GLP-1–secreting L cells in the gut, hopefully limiting any untoward side effects.

When translating animal studies into clinical studies, certain species-specific variations should be considered. It has been reported that SSTR5 is much more abundant in β cells from humans than from rodents (23, 51, 52). In line with that, a human study using the SS agonist pasireotide, with a high affinity for SSTR5, resulted in both decreased insulin and GLP-1 levels (53). Targeting both GLP-1 and insulin secretion with a SSTR5 antagonist could perhaps be an advantage in the treatment of hyperglycemia in humans.

## Methods

### Animals.

Male and female C57BL/6JRj mice (9–12 weeks of age; males, 24–30 g; females, 18–23 g) were purchased from Janvier Labs. Male GLP-1R–knockout (*Glp-1r^–/–^*) animals and WT littermates (*Glp-1r^+/+^*) (9–16 weeks of age, 24–30 g) were developed in our laboratory and characterized previously (54). Male DIO mice and controls (27–31 weeks of age, DIO, 46–59 g; controls, 30–36 g) were purchased from Taconic. Male SSTR5-knockout mice (*Sstr5^–/–^)* and *Sstr5^+/+^* littermates (18 weeks of age, 34–40 g) were a gift from Eli Lilly Research Laboratories (Lilly), originally obtained from Taconic. C57BL/6JRj and *Glp-1r^–/–^* mice were housed 2–8 mice per cage, whereas the *Sstr5^–/–^* mice and *Sstr5^+/+^* were housed 1–3 per cage. The DIO and control mice were housed 4–6 mice per cage. All mice were under a 12-hour-light/dark cycle with free access to standard rodent chow and water. The DIO mice had free access to a high-fat diet (catalog D12492, 60 % kcal% fat, Research Diets Inc.) instead of standard chow.

### Compounds.

The following compounds were purchased from Bachem: SSTR2 antagonist (PRL-2915, catalog H-6056), Ex9–39 (catalog H-8740), GLP-1 (catalog H-6795), and Bombesin (catalog H-2155). Dimethyl sulfoxide (DMSO, CAS no. 67-68-5), 2-hydroxypropyl β-cyclodextrin (HPCD, catalog H107), and arginine (catalog A5131) were purchased from Sigma-Aldrich. The SSTR5 antagonist (compound B, 6-[[1-[(4-Chloro-3,5-diethoxyphenyl)methyl]-4-piperidinyl]amino]-3-pyridinecarboxylic acid; hydrochloride) was a gift from F. Hoffmann-La Roche Ltd. (22). The DPP-4i valine pyrrolidide was a gift from Novo Nordisk A/S.

### Perfusion of the proximal small intestine and pancreas in mice.

Male C57BL/6JRj, DIO/control mice, or *Sstr5^–/–^ /* *Sstr5^+/+^* were anesthetized with an i.p. injection of Ketamine/Xylazine (0.1 mL/20 g) (90 mg/kg Ketamine, Ketaminol Vet, MSD Animal Health; 10 mg/kg Xylazine, Rompun Vet., Bayer Animal Health). The perfusion setup and procedures have been described previously (17, 31, 55). In short, the colon and the distal part of the intestine were removed leaving 12–15 cm of the proximal small intestine for perfusions. When performing the pancreas perfusion, the entire intestine was removed, except for the most proximal part of the duodenum, which shares vessels with the pancreas. Next, for both operations, the spleen and stomach were tied off and removed and the kidneys were ligated. For the intestinal perfusions, a tube was placed in the proximal opening of the intestine, allowing luminal perfusion of 37°C isotonic saline (0.9%) NaCl, at a rate of 0.035 mL/min. Hereafter a catheter (BD Insyte Autoguard, 24 GA 0.75 IN, 0.7 × 19 mm) was placed in the abdominal part of the aorta for vascular perfusion of the intestine and pancreas via the celiac and the superior mesenteric arteries. A similar catheter was placed in the portal vein and the effluent was collected every minute using a fraction collector. When the catheters were in place, the mice were euthanized by perforation of the diaphragm. The intestine was perfused at a flow rate of 2.5 mL/min and the pancreas with 1.5 mL/min using a modified Krebs Ringer bicarbonate buffer. The buffer was pH adjusted to approximately 7.5 and contained 0.1% BSA (Merck KGaA), 5% Dextran T-70 (Dextran Products Limited), 3.5 mmol/L glucose, and 5 mmol/L pyruvate, 5 mmol/L fumarate, and 5 mmol/L glutamate; for gut perfusions, the following were additionally added to the buffer: 10 μmol/L 3-isobutyl-1-methylxanthine (IBMX) and Vamin (a mixture of essential and nonessential amino acids; Fresenius Kabi). In a subset of the pancreas perfusions, the concentration of glucose in the buffer was increased from 3.5 to 15 mM after 40 minutes. During the experiment, the perfusion medium was heated to 37°C and gassed with a 95% O_2_/5% CO_2_ mixture. After an equilibration period of 30 minutes, the experiment was initiated.

The perfused intestine was stimulated with 20% (w/v) glucose from the luminal side alone or in combination with intra-arterial infusions of 1 μM SSTR2a, SSTR5a, or Ex9–39. The chosen concentration was based on in vitro data showing, for both antagonists, half maximal inhibitory concentrations (IC_50_) in the nanomolar range for their respective receptors (IC_50_ for SSTR2a 355 nM, 80 nM for SSTR5a and 12 nM for the GLP-1R) as well as on dose-response experiments carried out in the perfused proximal mouse intestine, as published previously (17, 56).

### Oral glucose tolerance tests.

Male and female C57BL/6JRj, male *Glp-1r^–/–^* and *Glp-1r^+/+^*, male *Sstr5^–/–^* and *Sstr5^+/+^*, and male DIO and control mice were fasted for 5–6 hours (0800–1300/1400 hours) with free access to water. The following compounds were administered by s.c. injection 15 minutes before an oral glucose load (0.004 mL/g, 2 g/kg body weight, 50% w/v dissolved in 0.9% NaCl). SSTR5a dissolved in PBS + 1.5% DMSO was given in a dose of 4 mg/kg (or 8 mg/kg for the combination study with DPP-4i), SSTR2a dissolved in PBS + 1.5% DMSO in a dose of 4 mg/kg, Ex9–39 dissolved in milliQ water + 1.5% DMSO in a dose of 4 mg/kg, valine pyrrolidide dissolved in PBS 120 mg/kg, and GLP-1 dissolved in milliQ water + 1% BSA in a dose of 30 nmol/kg. Vehicle groups received PBS + 1.5% DMSO. In DIO and control mice, the SSTR2 and SSTR5 antagonists were administered by oral gavage at a dose of 50 mg/kg and dissolved in a 30% HPCD and 70% milliQ water solution. Oral gavage of the antagonists was applied 30 minutes before the oral glucose load. Blood glucose concentrations were measured in tail blood (~2 μL) with a handheld glucometer (Accu-Chek Mobile, catalog 05874149001; Roche Diagnostics). For hormone measurements, larger blood samples (~75 μL) were collected from the retrobulbar plexus using EDTA-coated capillary tubes (Micro Haematocrit Tubes, Ref. 167313 Vitrex Medical A/S) at times 0, 15, 30, and 60 minutes or –15, 0, 15, and 30 minutes. Blood was transferred to Eppendorf tubes and centrifuged at 3000*g* for 10 minutes. Plasma was transferred to PCR tubes and stored at –80°C until analysis.

### Biochemical measurements for perfusion studies.

Effluent samples from the perfused intestine and pancreas were measured by validated in-house RIAs using antibodies targeting GLP-1, glucagon, insulin, and SS. The GLP-1 antibody (ab 89390) targets the amidated C-terminus of the peptide, thereby quantifying total GLP-1 (intact as well as N-terminally truncated forms of GLP-1) (57). Glucagon was measured using a C-terminally directed antibody (ab 4305) (58) and insulin was measured using an antibody raised against porcine insulin, which cross-reacts strongly with mouse, human, and rat insulin (ab 2006-3) (59). SS was measured with an antibody targeting both isoforms of SS (SS-14 and SS-28, ab 1758) (60).

### Biochemical measurements for in vivo studies.

Plasma concentrations of insulin and GLP-1 were quantified using ELISAs from Mercodia (catalog 10-1247-01 and 10-1278-01, respectively) and carried out according to the manufacturer’s protocols.

### Statistics.

All data are presented as the mean ± SEM, and differences resulting in *P* < 0.05 were considered significant. In the perfusion studies, changes were evaluated based on either the mean incremental hormone output (subtraction of the preceding mean 5-minute baseline output from the mean stimulation period plus 5-minute poststimulation period, due to the often delayed response previously observed, ref. 17) or on total mean output. When data from 2 consecutive stimulations in the same animal were compared, significance was tested by paired 2-tailed *t* test; when testing responses from 2 different types of mice (i.e., WT versus knockout mice), significance was tested by unpaired 2-tailed *t* test. Correlation analysis was based on the average output each minute from 1 minute to 100 minutes or from 1 minute to 40 minutes. Statistical evaluations in all in vivo studies are based on the iAUC from oral glucose gavage or i.p. administration (time 0 min) until the end of the experiment. Significance was evaluated by 1-way ANOVA followed by the Holm-Sidak post hoc analysis to correct for multiple testing. Time-dependent changes in 2 groups were evaluated by 2-way ANOVA followed by Tukey post hoc analysis to correct for multiple testing. Calculations and graphs were made in GraphPad Prism 6.

### Study approval.

All mice were used and kept in accordance with the recommendations of the NIH (publication number 85-23), and experiments were carried out with permission from the Danish Animal Experiments Inspectorate (2013-15-2934-00833 and 2018-15-0201-01397).

## Author contributions

SLJ, NJWA, JP, CFD, and JJH conceived of and designed the research. SLJ, JAW, KDG, and JEH performed the in vivo experiments. SLJ performed and designed the perfusion experiments. SLJ, NJWA, JAW, KDG, JEH, TBF, HK, and REM analyzed and interpreted the results and SLJ drafted the manuscript. NJWA, JAW, KDG, JEH, TBF, HK, JP, CFD, REM, and JJH edited and revised the manuscript. All authors approved the final version of the manuscript.

## Supplementary Material

Supplemental data

## Version 1 01/12/2021

In-Press Preview

## Version 2 02/22/2021

Electronic publication

## References

[1] Adriaenssens AE (2018). Distribution and stimulus secretion coupling of enteroendocrine cells along the intestinal tract. Compr Physiol.

[2] Holst JJ (2013). Enteroendocrine secretion of gut hormones in diabetes, obesity and after bariatric surgery. Curr Opin Pharmacol.

[3] Muscogiuri G (2018). Gut: a key player in the pathogenesis of type 2 diabetes?. Crit Rev Food Sci Nutr.

[4] Holst JJ (2019). The incretin system in healthy humans: the role of GIP and GLP-1. Metabolism.

[5] Holst JJ (2011). Loss of incretin effect is a specific, important, and early characteristic of type 2 diabetes. Diabetes Care.

[6] Nauck MA, Meier JJ (2016). The incretin effect in healthy individuals and those with type 2 diabetes: physiology, pathophysiology, and response to therapeutic interventions. Lancet Diabetes Endocrinol.

[7] Drucker DJ (1987). Glucagon-like peptide I stimulates insulin gene expression and increases cyclic AMP levels in a rat islet cell line. Proc Natl Acad Sci U S A.

[8] Holst JJ (1987). Truncated glucagon-like peptide I, an insulin-releasing hormone from the distal gut. FEBS Lett.

[9] Nauck M (1986). Reduced incretin effect in type 2 (non-insulin-dependent) diabetes. Diabetologia.

[10] Nauck MA (1998). Normalization of fasting glycaemia by intravenous GLP-1 ([7–36 amide] or [7–37]) in type 2 diabetic patients. Diabet Med.

[11] Todd JF (1998). Subcutaneous glucagon-like peptide-1 improves postprandial glycaemic control over a 3-week period in patients with early type 2 diabetes. Clin Sci (Lond).

[12] Nauck MA (1996). Effects of subcutaneous glucagon-like peptide 1 (GLP-1 [7–36 amide]) in patients with NIDDM. Diabetologia.

[13] Nauck MA (1993). Normalization of fasting hyperglycaemia by exogenous glucagon-like peptide 1 (7–36 amide) in type 2 (non-insulin-dependent) diabetic patients. Diabetologia.

[14] Herman GA (2006). Effect of single oral doses of sitagliptin, a dipeptidyl peptidase-4 inhibitor, on incretin and plasma glucose levels after an oral glucose tolerance test in patients with type 2 diabetes. J Clin Endocrinol Metab.

[15] Hansen L (2000). Somatostatin restrains the secretion of glucagon-like peptide-1 and -2 from isolated perfused porcine ileum. Am J Physiol Endocrinol Metab.

[16] Hansen L (2004). Glucagon-like peptide-1 secretion is influenced by perfusate glucose concentration and by a feedback mechanism involving somatostatin in isolated perfused porcine ileum. Regul Pept.

[17] Jepsen SL (2019). Paracrine crosstalk between intestinal l- and D-cells controls secretion of glucagon-like peptide-1 in mice. Am J Physiol Endocrinol Metab.

[18] Martin PA, Faulkner A (1996). Effects of somatostatin-28 on circulating concentrations of insulin and gut hormones in sheep. J Endocrinol.

[19] Patel YC (1995). The somatostatin receptor family. Life Sci.

[20] Schonbrunn A (1978). Characterization of functional receptors for somatostatin in rat pituitary cells in culture. J Biol Chem.

[21] Yamada Y (1992). Cloning and functional characterization of a family of human and mouse somatostatin receptors expressed in brain, gastrointestinal tract, and kidney. Proc Natl Acad Sci U S A.

[22] Sprecher U (2010). Novel, non-peptidic somatostatin receptor subtype 5 antagonists improve glucose tolerance in rodents. Regul Pept.

[23] Farb TB (2017). Regulation of endogenous (male) rodent GLP-1 secretion and human islet insulin secretion by antagonism of somatostatin receptor 5. Endocrinology.

[24] Briere DA (2018). Mechanisms to elevate endogenous GLP-1 beyond injectable GLP-1 analogs and metabolic surgery. Diabetes.

[25] Hirose H (2017). Discovery of novel 5-oxa-2,6-diazaspiro[3.4]oct-6-ene derivatives as potent, selective, and orally available somatostatin receptor subtype 5 (SSTR5) antagonists for treatment of type 2 diabetes mellitus. Bioorg Med Chem.

[26] Strowski MZ (2003). Somatostatin receptor subtype 5 regulates insulin secretion and glucose homeostasis. Mol Endocrinol.

[27] Wang XP (2005). SSTR5 ablation in islet results in alterations in glucose homeostasis in mice. FEBS Lett.

[28] Tirone TA (2003). Pancreatic somatostatin inhibits insulin secretion via SSTR-5 in the isolated perfused mouse pancreas model. Pancreas.

[29] Moss CE (2012). Somatostatin receptor 5 and cannabinoid receptor 1 activation inhibit secretion of glucose-dependent insulinotropic polypeptide from intestinal K cells in rodents. Diabetologia.

[30] Strowski MZ (2000). Somatostatin inhibits insulin and glucagon secretion via two receptors subtypes: an in vitro study of pancreatic islets from somatostatin receptor 2 knockout mice. Endocrinology.

[31] Orgaard A, Holst JJ (2017). The role of somatostatin in GLP-1-induced inhibition of glucagon secretion in mice. Diabetologia.

[32] Svendsen B, Holst JJ (2016). Regulation of gut hormone secretion. Studies using isolated perfused intestines. Peptides.

[33] Bak MJ (2014). Specificity and sensitivity of commercially available assays for glucagon-like peptide-1 (GLP-1): implications for GLP-1 measurements in clinical studies. Diabetes Obes Metab.

[34] Windelov JA (2017). Why is it so difficult to measure glucagon-like peptide-1 in a mouse?. Diabetologia.

[35] Hocart SJ (1999). Highly potent cyclic disulfide antagonists of somatostatin. J Med Chem.

[36] Ludvigsen E (2004). Expression and distribution of somatostatin receptor subtypes in the pancreatic islets of mice and rats. J Histochem Cytochem.

[37] Wang XP (2004). Double-gene ablation of SSTR1 and SSTR5 results in hyperinsulinemia and improved glucose tolerance in mice. Surgery.

[38] Fagan SP (1998). Insulin secretion is inhibited by subtype five somatostatin receptor in the mouse. Surgery.

[39] Yue JT (2012). Somatostatin receptor type 2 antagonism improves glucagon and corticosterone counterregulatory responses to hypoglycemia in streptozotocin-induced diabetic rats. Diabetes.

[40] Karimian N (2013). Somatostatin receptor type 2 antagonism improves glucagon counterregulation in biobreeding diabetic rats. Diabetes.

[41] Taleb N, Rabasa-Lhoret R (2016). Can somatostatin antagonism prevent hypoglycaemia during exercise in type 1 diabetes?. Diabetologia.

[42] Leclair E (2016). Glucagon responses to exercise-induced hypoglycaemia are improved by somatostatin receptor type 2 antagonism in a rat model of diabetes. Diabetologia.

[43] Singh V (2007). Somatostatin receptor subtype-2-deficient mice with diet-induced obesity have hyperglycemia, nonfasting hyperglucagonemia, and decreased hepatic glycogen deposition. Endocrinology.

[44] Chambers AP (2017). The role of pancreatic preproglucagon in glucose homeostasis in mice. Cell Metab.

[45] Karlsson S, Ahren B (1991). Insulin and glucagon secretion in swimming mice: effects of adrenalectomy and chemical sympathectomy. J Auton Nerv Syst.

[46] Thorens B (2014). Neural regulation of pancreatic islet cell mass and function. Diabetes Obes Metab.

[47] Aaboe K (2010). Twelve weeks treatment with the DPP-4 inhibitor, sitagliptin, prevents degradation of peptide YY and improves glucose and non-glucose induced insulin secretion in patients with type 2 diabetes mellitus. Diabetes Obes Metab.

[48] Whalen KA (2019). Targeting the somatostatin receptor 2 with the miniaturized drug conjugate, PEN-221: a potent and novel therapeutic for the treatment of small cell lung cancer. Mol Cancer Ther.

[49] Wada H (2016). Expression of somatostatin receptor type 2A and PTEN in neuroendocrine neoplasms is associated with tumor grade but not with site of origin. Endocr Pathol.

[50] Hernandez Vargas S (2019). Specific targeting of somatostatin receptor subtype-2 for fluorescence-guided surgery. Clin Cancer Res.

[51] Kumar U (1999). Subtype-selective expression of the five somatostatin receptors (hSSTR1-5) in human pancreatic islet cells: a quantitative double-label immunohistochemical analysis. Diabetes.

[52] Singh V (2007). Characterization of somatostatin receptor subtype-specific regulation of insulin and glucagon secretion: an in vitro study on isolated human pancreatic islets. J Clin Endocrinol Metab.

[53] Henry RR (2013). Hyperglycemia associated with pasireotide: results from a mechanistic study in healthy volunteers. J Clin Endocrinol Metab.

[54] Balk-Moller E (2020). Glucagon-like peptide 1 and atrial natriuretic peptide in a female mouse model of obstructive pulmonary disease. J Endocr Soc.

[55] Jepsen SL (2019). Ghrelin does not directly stimulate secretion of glucagon-like peptide-1. J Clin Endocrinol Metab.

[56] Svendsen B (2018). Insulin secretion depends on intra-islet glucagon signaling. Cell Rep.

[57] Orskov C (1994). Tissue and plasma concentrations of amidated and glycine-extended glucagon-like peptide I in humans. Diabetes.

[58] Orskov C (1991). Proglucagon products in plasma of noninsulin-dependent diabetics and nondiabetic controls in the fasting state and after oral glucose and intravenous arginine. J Clin Invest.

[59] Brand CL (1995). Role of glucagon in maintenance of euglycemia in fed and fasted rats. Am J Physiol.

[60] Hilsted L, Holst JJ (1982). On the accuracy of radioimmunological determination of somatostatin in plasma. Regul Pept.

