# Supplementary material for: Antagonizing somatostatin receptor subtype 2 and 5 reduces blood glucose in a gut- and GLP-1R–dependent manner
Source: JCI Insight. 2021 Feb 22;6(4):e143228. doi: 10.1172/jci.insight.143228 (PMC7934931; doi:10.1172/jci.insight.143228)
Supplement: Supplemental data [file jciinsight-6-143228-s021.pdf]

Supplementary figures

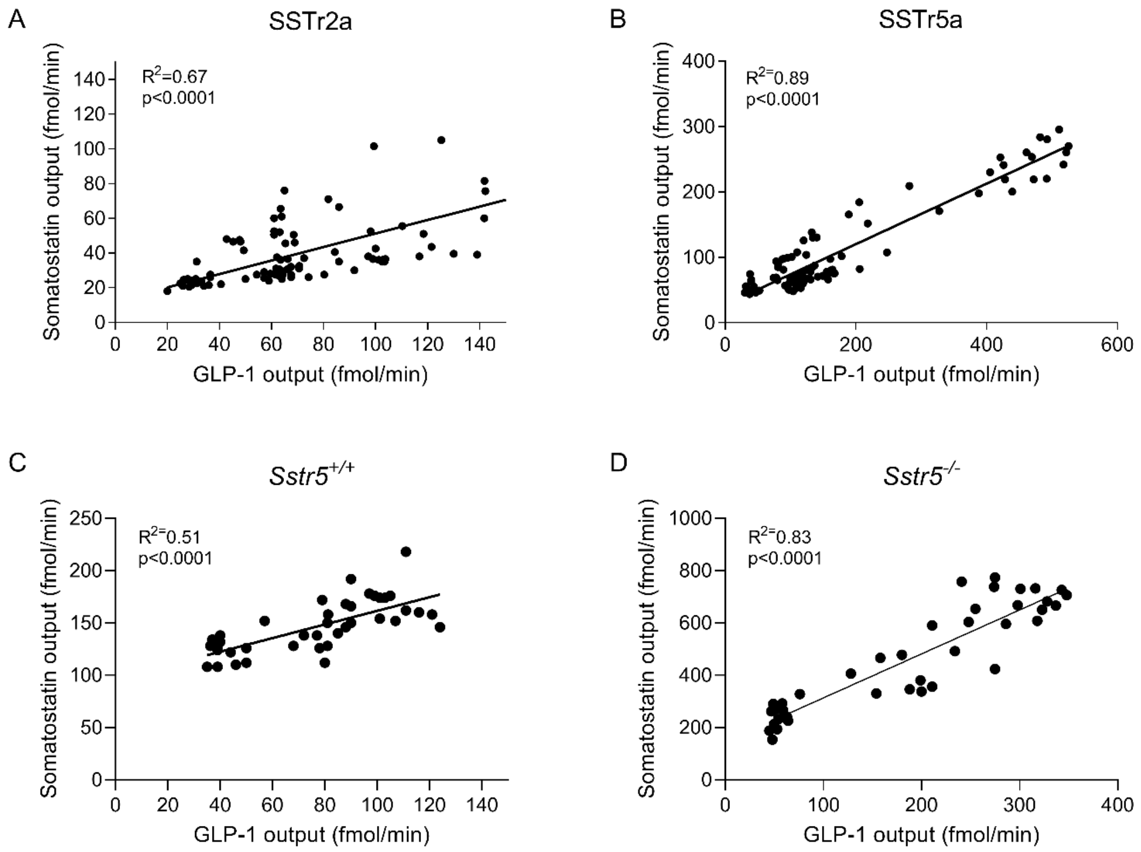

Supplementary fig. S1 correlation analysis between somatostatin and GLP-1 output. Correlation analysis are based on average output each minute from somatostatin and GLP-1 during the perfusion of the proximal small intestine. A) Average output each minute from 1-100 min in C57BL/6JRj mice of somatostatin and GLP-1 output in perfusion experiments where glucose or glucose + SSTR2a was given (shown in Fig. 1A-D). GLP-1 average of n=8, SS based on n=5. B) Same as A, but for SSTR5a infusion, average of n=6 for both hormones (shown in fig 1 E-H). C) Average output each minute from 1-40 min in $Sstr5^{-/-}$ of GLP-1 and somatostatin n=5 for both hormones (shown in fig. 1 I-K). D) Same as C, but in $Sstr^{-/-}$ mice, n=5 for both hormones (shown in fig. 1 I-K).

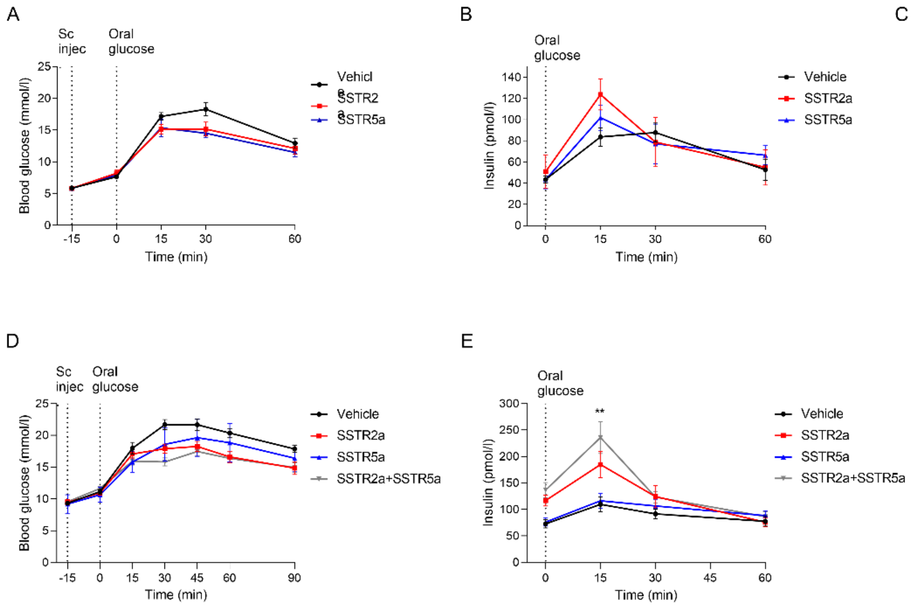

Supplementary fig. S2. SSTr2a and SSTr5a decrease blood glucose *in vivo* in female mice and combining the antagonists in male mice further improves glucose tolerance. Female mice received vehicle (black), 4 mg/kg SSTR2a (red) or SSTR5a (blue) by sc injection 15 minutes before an oral glucose load. A) Plasma blood glucose levels (mmol/l), n=8. B) Plasma insulin levels (pmol/l), n=8. C) Total GLP-1 plasma levels (pmol/l), n=8. D) Male mice received vehicle (black), 4 mg/kg SSTR2a (red), SSTR5a (blue) or SSTR2a+ SSTR5a in combination (grey) by sc injection 15 minutes before an oral glucose load. D) Plasma blood glucose levels (mmol/l), n=12. E) Plasma insulin levels (pmol/l), n=12. Data are presented as mean ± SEM. Significance at specific time points was assessed by two-way ANOVA followed by Tukey post hoc analysis to correct for multiple testing.

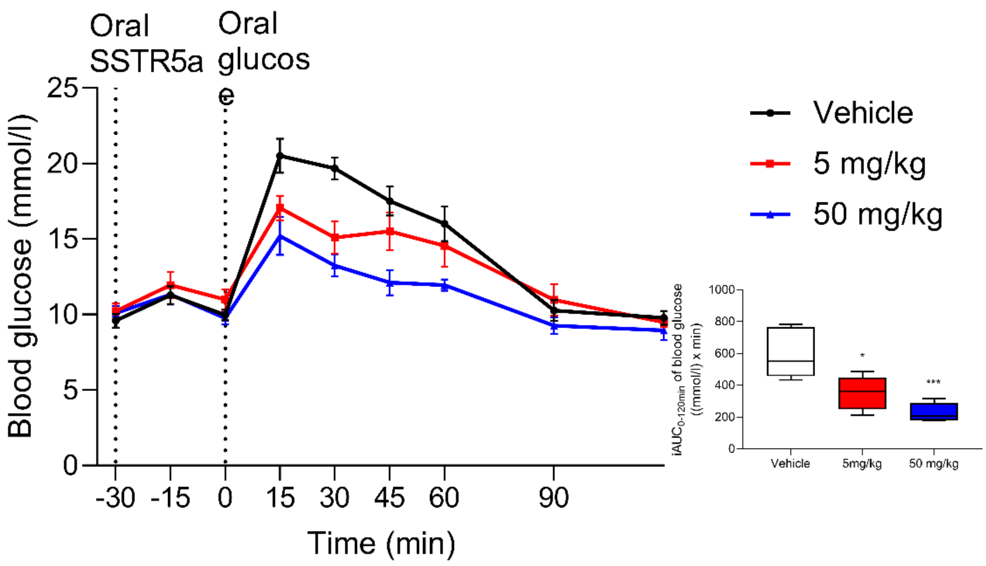

Supplementary fig. S3. Oral gavage of SSTR5a decrease blood glucose in a dose dependent manner. Male mice received vehicle (black), 5 mg/kg SSTR5a (red) or 50 mg/kg SSTR5a (blue) by oral gavage 30 minutes before an oral glucose load. Data are presented as mean ± SEM, n=5-6. Statistical significance was based on iAUC from 0-120 min and tested by one-way ANOVA followed by the Tukey post hoc analysis to correct for multiple testing, *p<0.05, ***p<0.001. The box plots show the median and 25th and 75th percentiles, and the whiskers represent the smallest and highest value.

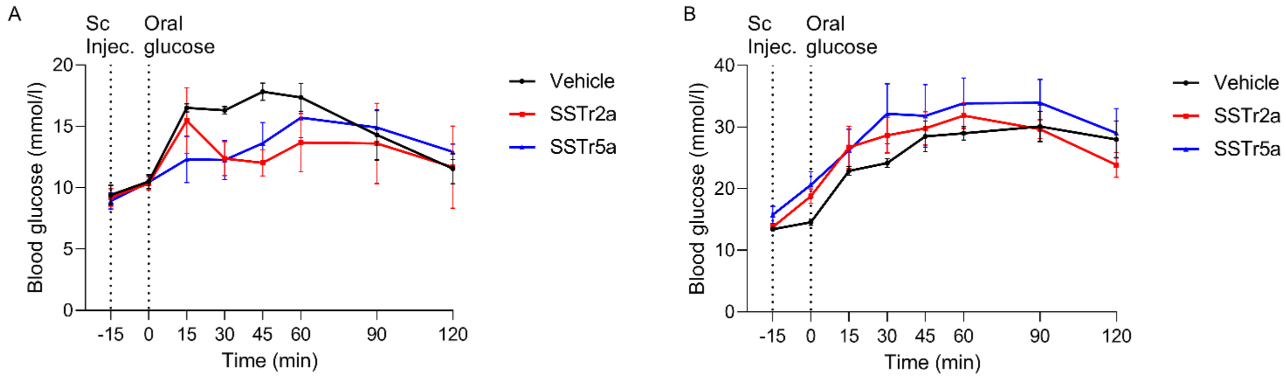

Supplementary fig. S4. Sc injection of SSTr2a and SSTr5a lowers blood glucose in control mice but have no effect in DIO mice. Control and DIO mice received sc administration of vehicle (black), 8 mg/kg SSTr2a (red) or SSTr5a (blue) 15 min before oral glucose. A) Plasma blood glucose levels (mmol/l) in control mice, n=4 B) Plasma blood glucose levels (mmol/l) in DIO mice, n=5-6. Data are presented as means ± SEM.
